# Heparin-induced DRESS syndrome in a paediatric patient and successful anaesthetic management in cardiovascular bypass surgery: case report

**DOI:** 10.1186/s13019-024-02722-x

**Published:** 2024-04-17

**Authors:** Laura Peña-Blanco, Laura Gutiérrez-Soriano, Félix Ramón Montes, Andrea Barragán-Méndez, Susana Beltrán-Villegas, Juan José López-Reyes, Carlos A. Villa-Hincapié, Juan Pablo Umaña

**Affiliations:** 1 Anesthesiology Department, Fundación Cardioinfantil-Instituto de Cardiología, Bogotá, Colombia; 2 Universidad del Rosario, Bogotá, Colombia; 3 Department of Cardiovascular Surgery, Fundación Cardioinfantil-Instituto de Cardiología, Bogotá, Colombia

**Keywords:** Drug hypersensitivity syndrome, Heparin, Eosinophilia, Cardiopulmonary bypass, Bivalirudin

## Abstract

**Background:**

Drug Reaction with Eosinophilia and Systemic Symptoms (DRESS) Syndrome is a severe adverse drug reaction marked by delayed hypersensitivity reactions causing skin and systemic complications. DRESS diagnosis is challenging due to the variety of clinical presentations and symptom overlap with other conditions. The perioperative period in these patients requires precise pharmacological strategies to prevent complications associated with this syndrome. The treatment of DRESS induced by unfractionated heparin during cardiopulmonary bypass (CPB) surgery presents some challenges that must be considered when selecting an anticoagulant to avoid side effects. In this case, bivalirudin, a direct thrombin inhibitor, is indicated as an alternative to heparin in patients undergoing CPB. However, in contrast to heparin/protamine, there is no direct reversal agent for bivalirudin.

**Case presentation:**

We report the case of an 11-year-old male diagnosed with native aortic valve endocarditis and thrombosis in his left lower extremity. During valvular replacement surgery, systemic unfractionated heparin was administered. Postoperatively, the patient developed fever, eosinophilia and pruritic rash. Warm shock and elevated alanine transaminase (ALT) and aspartate transaminase (AST) levels followed, leading to the diagnosis of DRESS syndrome. Treatment with methylprednisolone resulted in complete resolution of symptoms. Seven years later, the patient was readmitted due to insufficient anticoagulation and a thrombus in the prosthetic aortic valve, presenting a recurrent DRESS episode due to the administration of unfractionated heparin, which was later replaced with low-molecular-weight heparin during hospitalization. Treatment with corticosteroids and antihistamines was initiated, resulting in the resolution of this episode. Ultimately, the patient required the Ross procedure. During this intervention the anticoagulation strategy was modified, unfractionated heparin was replaced with bivalirudin during the procedure and fondaparinux was administered during the postoperative period. This resulted in stable transaminases levels and no eosinophilia.

**Conclusion:**

The severity of DRESS Syndrome underscores the importance of early recognition, heightened monitoring, and a comprehensive approach tailored to each patient’s needs. This particular case highlights the significance of this approach and may have a substantial clinical impact since it provides alternatives to heparin, such as bivalirudin and fondaparinux, in the anticoagulation strategy of CPB for patients who have a hypersensibility reaction to this medication; thus, enhancing clinical outcomes by minimizing risks linked to adverse drug reactions.

## Background

Drug Reaction with Eosinophilia and Systemic Symptoms (DRESS) Syndrome is a severe, potentially life-threatening adverse drug reaction that affects both adults and children [1]. It is a delayed hypersensitivity reaction characterized by skin manifestations and the involvement of internal organs, particularly the skin, liver and hematologic system [2]. Early recognition and an accurate diagnosis of DRESS are essential for avoiding serious complications and providing appropriate treatment. However, due to the variability of its clinical presentation and the overlap of symptoms with other diseases, the diagnosis can be challenging. In addition, one of the challenges during the anaesthetic and perioperative period in these patients consists of preventing a drug hypersensibility reaction.

Treatment of DRESS syndrome triggered by unfractionated heparin during subsequent cardiopulmonary bypass (CPB) surgeries is a significant challenge, and alternative anticoagulants are needed to prevent adverse reactions. Heparin-induced DRESS is rare and has a limited number of reported cases. Although hypersensitivity reactions to heparin are uncommon but serious, they remain an important clinical concern in patients requiring CPB. Even though the use of bivalirudin, a reversible direct thrombin inhibitor, is unusual in our context, has emerged as an alternative to heparin in patients who undergo CPB.

## Case presentation

**Fig. 1:**
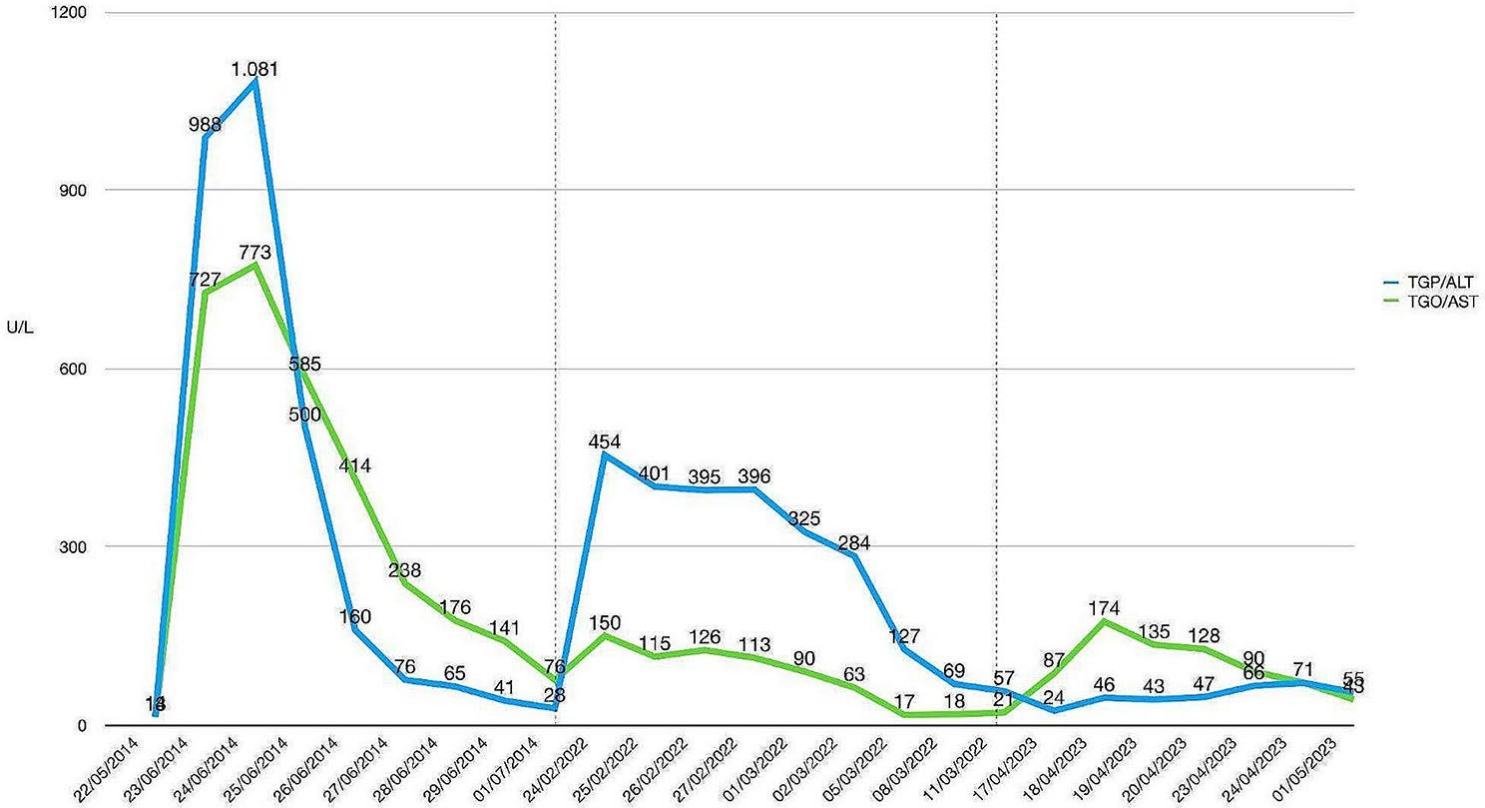
Behaviour of the aspartate aminotransferase (AST) and the alanine aminotransferase (ALT) during episodes of DRESS syndrome

**Fig. 2:**
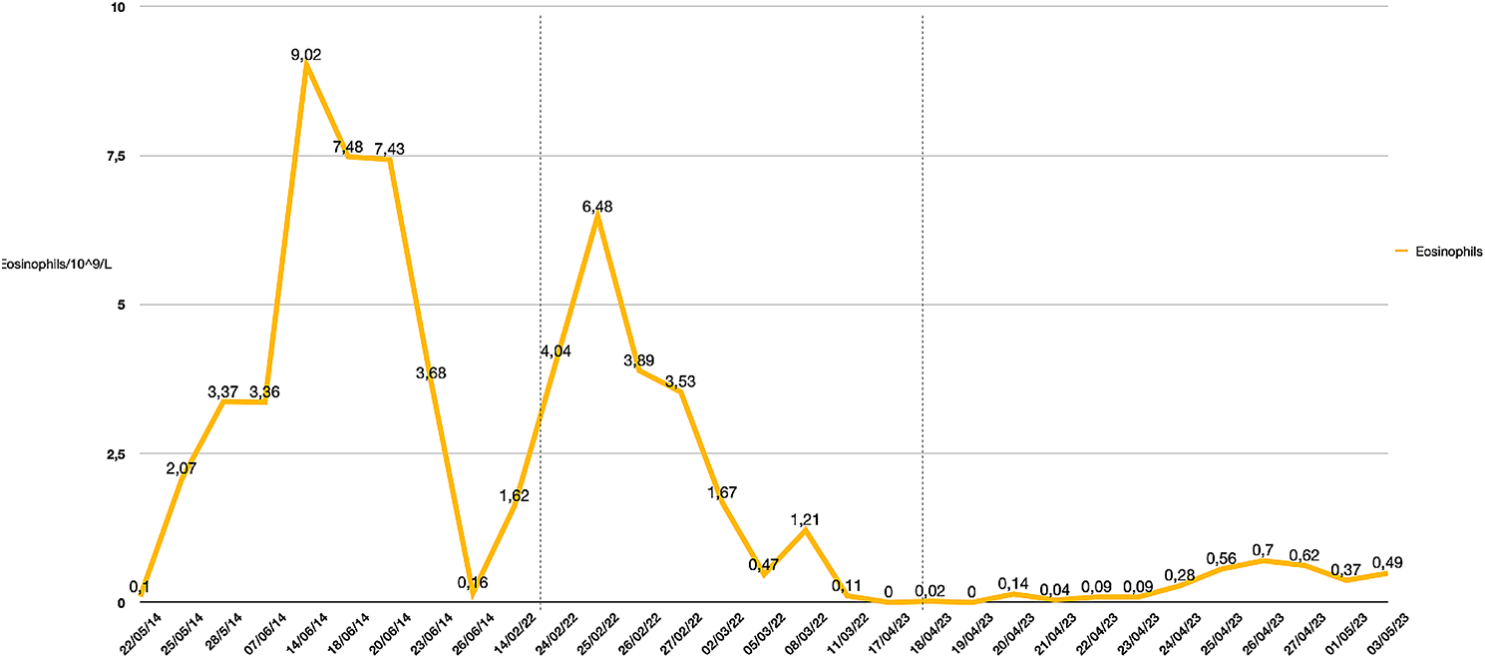
Behaviour of eosinophils during episodes of DRESS syndrome

**Fig. 3:**
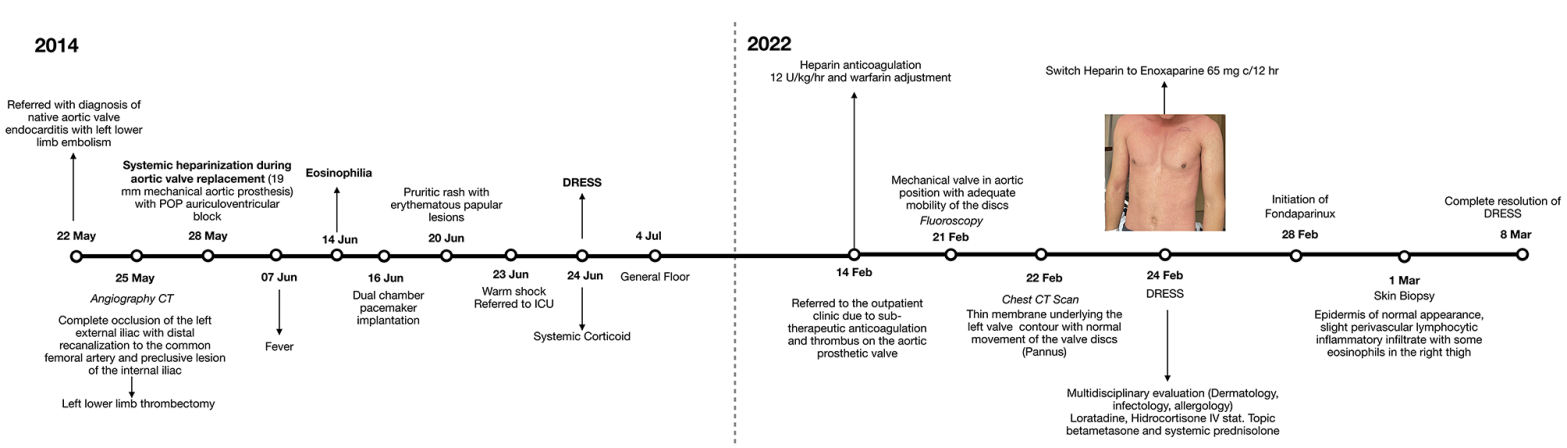
Sequence of events during episodes of DRESS syndrome

An 11-year-old male was referred to our institution for a mechanical valvular replacement surgery on CPB due to a diagnosis of native aortic valve endocarditis and embolism in the left lower extremity. CPB needed anticoagulation with unfractionated heparin. On postoperative day 10, the patient developed fever and increased eosinophil levels. This eosinophilia increased until it reached 9.02 × 10^9^/L eosinophils on postoperative day 17, accompanied by a pruritic rash with erythematous papular lesions on postoperative day 23. During the following days, the patient experienced warm shock along with an abrupt elevation of ALT and AST, with levels peaking at 1081 U/L and 773 U/L, respectively (Fig. 1). Based on the clinical and laboratory findings, the patient was diagnosed with DRESS syndrome. Methylprednisolone treatment was initiated, and a significant decrease in transaminases and eosinophil levels was observed during the following week, reaching a count of 0.16 × 10^9^/L eosinophils (Fig. 2). Finally, the patient was transferred to the general ward for hospitalization and discharged upon complete resolution of the condition. (Fig. 3)

The patient was readmitted 7 years later due to the presence of a thrombus in the prosthetic aortic valve with signs of valve dysfunction with altered mobility and stenosis of one of the mechanical leaflets secondary to subtherapeutic levels of INR. Anticoagulation therapy was initiated with unfractionated heparin. Ten days after admission, the patient developed an extensive erythematous-edematous plaque on the anterior and dorsal trunk, along with elevated transaminases (ALT 454 U/L and AST 150 U/L) and eosinophilia (6.48 × 10^9^/L eosinophils) (Figs. 1 and 2). Due to the patient’s history, a recurrent case of DRESS syndrome was suspected, possibly triggered by the use of unfractionated heparin. Consequently, unfractionated heparin was discontinued, and low-molecular-weight heparin was initiated as an alternative. Treatment with hydrocortisone, oral prednisone, loratadine and topical betamethasone was established (Fig. 3).

Subsequently, the patient presented an increase in erythema and the appearance of edematous morbilliform plaques affecting approximately 50% of the body surface, including the face, neck and limbs. A possible cross-reaction was considered and enoxaparin was discontinued; this was replaced with fondaparinux. The patient continued to receive topical and intravenous antihistamines. After starting treatment, transaminases and eosinophils gradually decreased. Twelve days after initiation of the treatment, there was a complete resolution of DRESS syndrome symptoms. Considering the current stability of cardiovascular symptoms and the persistence of valvular dysfunction, regardless of optimum anticoagulation, a scheduled Ross surgery was indicated.

**Fig. 4:**
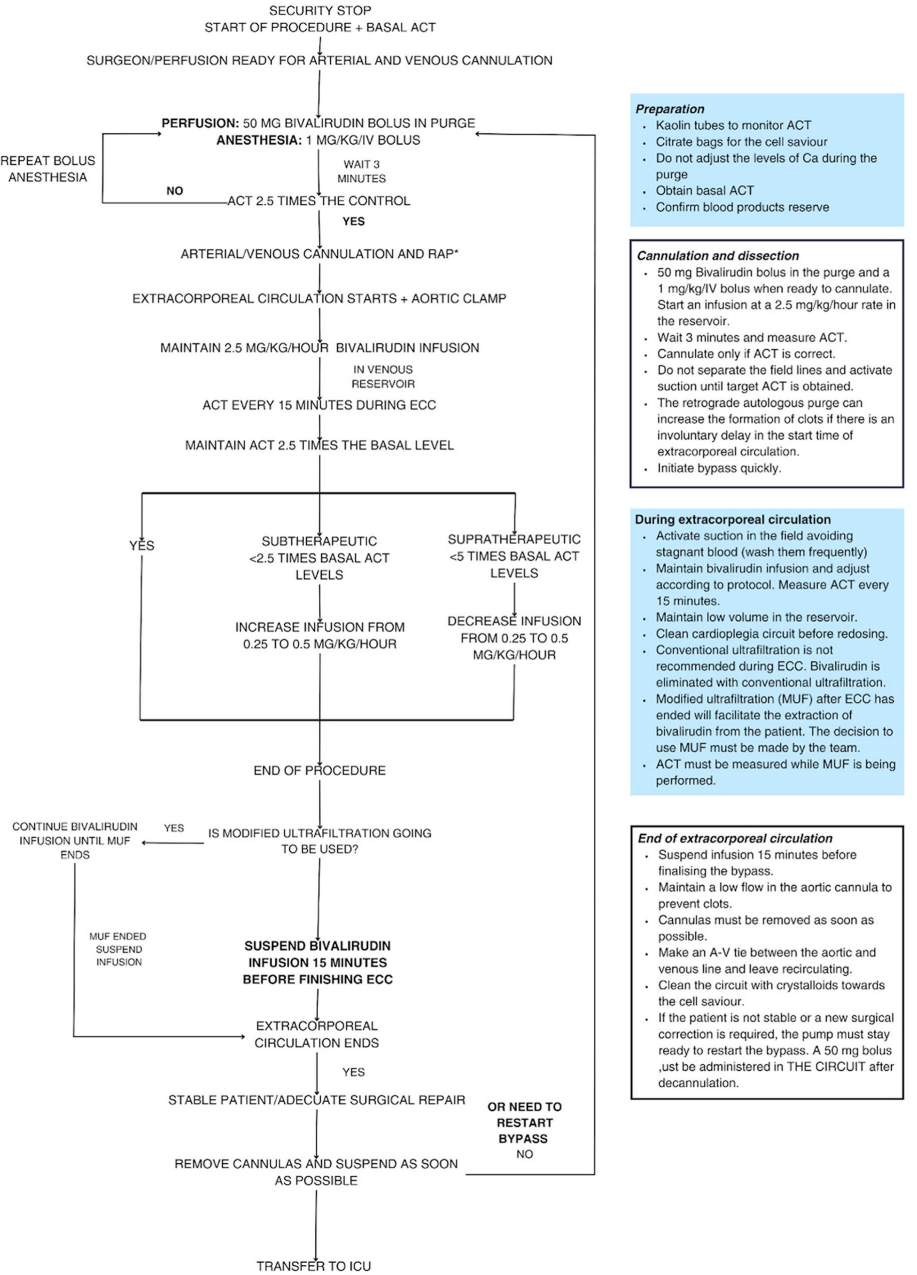
Bivalirudin use protocol

**Fig. 5:**
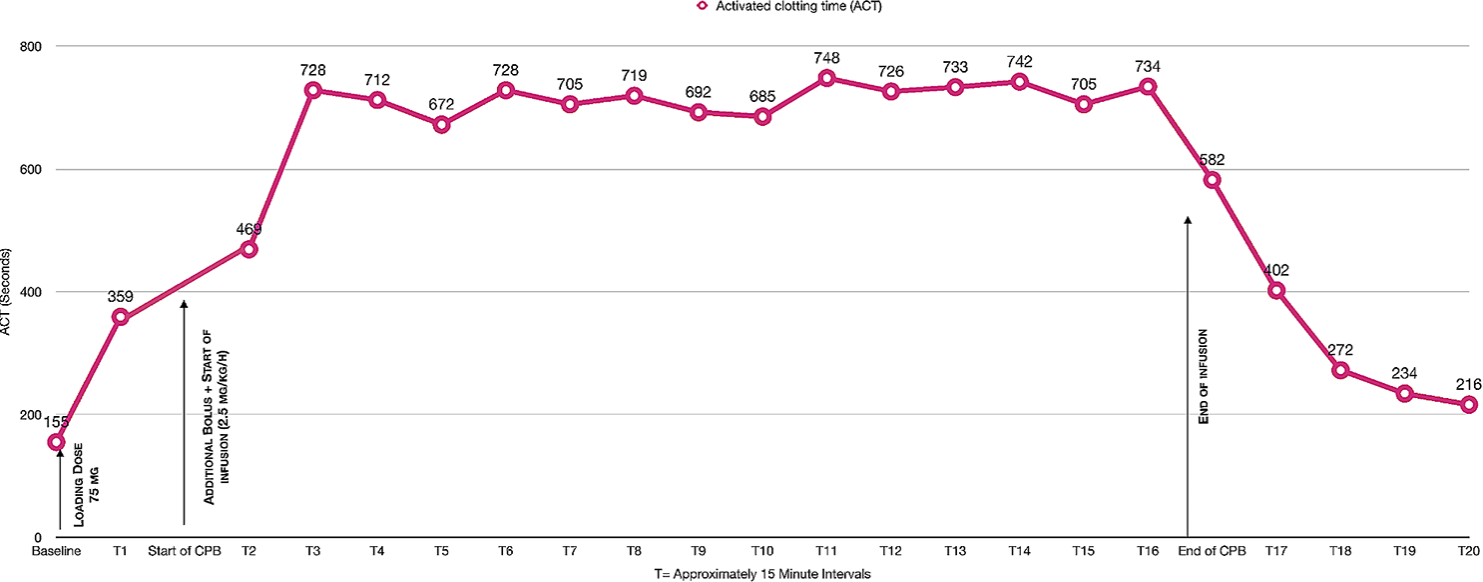
Behaviour of activated Clotting Time during bivalirudin protocol

Finally, in May 2023, 14 months after the resolution of DRESS, the patient underwent CPB surgery, requiring systemic anticoagulation with bivalirudin. The approach adhered to the institutional clinical practice guidelines concerning the administration protocol of bivalirudin and anaesthetic considerations. These guidelines recommend an initial dose of 1 mg/kg when the surgeon is ready to perform cannulation for CPB, an infusion rate of 2.5 mg/kg/h during extracorporeal circulation, and monitoring every 15 min. The goal of this regimen is to maintain an activated clotting time between 2.5 and 5 times the baseline value (Figs. 4 and 5). As ACT is altered after the procedure, it is important to note that the half-life of bivalirudin is approximately 25 min, depending on renal function, suggesting that the increase in ACT may be due to factors other than the administration of the drug.

The intraoperative complication was coagulopathy, which was effectively managed through comprehensive hemostasis techniques and the administration of four units of fresh frozen plasma, 20 units of cryoprecipitate, and two units of apheresis platelets, which is significantly higher than that used in a similar procedure performed under heparin anticoagulation at our institution.

Heparins were not used during the intraoperative and postoperative periods. Fondaparinux was used during the postoperative period as the anticoagulant of choice. During the hospitalization, no dermatologic manifestations were observed. The patient did not present eosinophilia and his ALT remained stable, with elevated AST levels coinciding with the period of vasoplegic shock. After appropriate management of concurrent infectious process (pneumonia), the patient was discharged with a satisfactory postoperative outcome.

## Discussion and conclusions

The incidence of DRESS varies depending on the type of medication and the patient’s immune status. In hospitalized patients, the incidence has been observed ranging from 2.18 to 40 cases per 100,000 admissions [3, 4]. Although it can affect individuals in any age group, most of the reported cases in the literature involve adults [2]. Despite its low incidence, DRESS has a high potential for morbidity and mortality, with mortality rates ranging from 3.8 to 10% [4].

Even though the exact pathophysiology of DRESS has not yet been fully elucidated, three key components in its development have been identified. The first is genetic susceptibility, which is related to certain human leukocyte antigen (HLA) alleles, that affect T lymphocyte responses. Second, alterations in the metabolic pathways of certain drugs have been observed, leading to the production of reactive oxygen metabolites that stimulate the immune response. Finally, virus reactivation has been implicated in triggering a T-cell-mediated inflammatory response and tissue damage. Medications associated with this reaction include aromatic anticonvulsants, sulphonamides, nonsteroidal anti-inflammatory drugs, and beta-lactam antibiotics, among others. However, in 10–20% of cases, the causative drug cannot be identified [5].

The clinical manifestations of DRESS syndrome appear weeks to months after initial exposure to the drug, with prodromal symptoms such as general malaise, pruritus and fever (between 38 and 40 °C). Later, they progress to cutaneous and systemic manifestations with a pruritic morbilliform rash that can spread rapidly and affect more than 50% of the body surface, facial edema, erythroderma and others [6, 7]. Hematologic involvement is also common, manifesting as eosinophilia, leucocytosis, the presence of atypical lymphocytes, thrombocytopenia, and anaemia [8, 9]. It should be noted that the liver is the organ most commonly affected, with increased liver enzymes, hepatomegaly, and jaundice, and severe liver necrosis and hepatic failure with coagulopathy may occur. Additionally, renal, pulmonary, cardiac, endocrine, neurological, and gastrointestinal system involvement is common [2, 10–12].

In the differential diagnosis, clinicians must exclude infectious causes, autoimmune disorders and severe drug-related skin reactions such as Stevens-Johnson syndrome (SJS) and toxic epidermal necrolysis (TEN). Differentiation between TEN/SJS and DRESS can be achieved by clinical and pathological assessment. Several factors, including the time of onset (which is prolonged in DRESS), the histology of the lesions, the extent of body surface involvement, the presence of facial angioedema, a positive Nikolsky’s sign in TEN patients, a shorter duration of symptoms in TEN and SJS patients, and additional clinical features help to differentiate these diseases [2].

The diagnosis of DRESS can be complex due to its diverse clinical presentation and the lack of specific tests for confirmation. It is based on clinical evaluation, history of drug exposure, and the latency period between drug exposure and symptom onset [2]. However, identifying the drug responsible can be challenging, especially when there are long latency periods or multiple drug use. Therefore, to determine the causative drug in DRESS cases, it is crucial to consider the timeline of drug administration, the interval between drug exposure and symptom onset, as well as the behaviour after drug withdrawal and the re-exposure effect [2].

In this particular case, anticoagulation for cardiovascular surgery represented a challenge due to the use of cardiopulmonary bypass and the natural tendency of blood to clot upon contact with foreign surfaces and the impossibility of using the usual anticoagulation strategy in this patient. As a result, it was necessary to implement special anticoagulation strategies during this procedure, using a drug other than unfractionated heparin, in this case bivalirudin, to prevent thrombus formation and reduce the risk of acute disseminated intravascular coagulation during bypass [13].

Heparins can cause various types of hypersensitivity reactions, particularly type IV cell-mediated reactions and type II antibody-mediated reactions (A type I hypersensitivity reaction is mediated by IgE antibodies that coat mast cells and basophils, this reaction develop after immediate contact with a free antigen. Type II hypersensitivity reactions include IgM and IgG cell binding, complement system activation and overall a cytotoxic reaction that result in cell lysis and phagocytosis. In contrast, a type IV hypersensitivity reaction involves a delayed pre-sensitised T-cell response) [14]. Rarely, immediate type I reactions can also occur. Its strong protein binding capacity seems to play an important pathogenic role. However, the allergens responsible for the different hypersensitivity reactions are still unknown [15].

According to the guidelines of the American Society of Extracorporeal Technology, the main contraindications to the use of heparin in the extracorporeal circulation are a history of heparin-induced thrombocytopenia (HIT) and known hypersensitivity to heparin. Although there are several alternatives to heparin, the lack of a rapidly reversible agent after withdrawal from CPB is the most challenging aspect. Among these alternatives, bivalirudin, a recombinant direct thrombin inhibitor, has been shown to be safe and effective, although its monitoring is more challenging than that of heparin [16]. Controlled trials suggest that bivalirudin provides adequate anticoagulation therapy in all patients, with similar outcomes in terms of mortality, 24-hour blood loss, overall transfusion rate, and surgical duration compared to heparin anticoagulation and protamine reversal. Although bivalirudin can increase the risk of excessive bleeding, it appears to be a safe and effective alternative to heparin and protamine reversal [16].

Regarding the complications of this management, postoperative bleeding after cardiac surgery with either bivalirudin or heparin as anticoagulants shares similar potential causes, including surgical bleeding, platelet issues, coagulation factor deficiencies, dilutional coagulopathy, and excessive fibrinolysis. Management strategies for excessive bleeding in both cases involve early re-exploration to address surgical causes and prevent cardiac tamponade. Suspected platelet problems are managed through transfusions, but diagnosing coagulation factor deficiencies can be challenging due to prothrombin time prolongation in bivalirudin-treated patients. In patients with significant bleeding, fresh frozen plasma transfusion may be considered [17]. Studies suggest higher blood loss in early postoperative periods for patients under bivalirudin, although differences in platelet transfusion and re-exploration rates did not show statistical significance. [18, 19].

## Conclusion

In conclusion, we present the case of a paediatric patient with a history of DRESS syndrome, possibly secondary to heparin, and a significant cardiovascular history requiring reintervention with the use of CPB. This case is unusual since heparin is not commonly classified as a causative agent of DRESS syndrome, which makes its diagnosis a challenge, especially when patients present conditions that require the use of different medications and interventions, as in the present case. During the management of these patient and their conditions, an early diagnosis, along with a multidisciplinary approach to address the hypersensitivity reactions and prevent complications, proved to be crucial. Furthermore, special anticoagulation strategies were necessary during the surgical intervention and postoperative period to avoid thrombotic complications. In this context, bivalirudin and fondaparinux have emerged as safe and effective alternatives to heparin. This case encourages working toward timely and accurate diagnoses that enable appropriate intervention, thereby preventing complications in these scenarios.

## Data Availability

Data sharing is not applicable to this article as no datasets were generated or analysed during the current study.

## Abbreviations

DRESS: Drug Reaction with Eosinophilia and Systemic Symptoms
AST: Aspartate aminotransferase
ALT: Alanine transaminase
HIT: heparin-induced thrombocytopenia
TEN: toxic epidermal necrolysis
SJS: Stevens-Johnson syndrome
CPB: cardiopulmonary bypass
